# PD-1 inhibitor combined with chemoradiotherapy in two cases of ovarian cancer brain metastases: a case report

**DOI:** 10.3389/fonc.2026.1690696

**Published:** 2026-05-28

**Authors:** Yanke Li, Yu Xia, Lei Wang, Xiaomei Liu, Zhenhua Du

**Affiliations:** 1 Department of Anus and Intestine Surgery, First Affiliated Hospital of China Medical University, Shenyang, Liaoning, China; 2 Department of Obstetrics and Gynecology, Shengjing Hospital of China Medical University, Shenyang, China

**Keywords:** brain metastases, immunotherapy, ovarian cancer, PD-1 inhibitor, tumor microenvironment

## Abstract

**Background:**

To investigate the clinical efficacy and potential mechanisms of immune checkpoint inhibitors (PD-1 inhibitors) combined with chemotherapy and targeted therapy in ovarian cancer patients with brain metastases, and to analyze the relationship between the number of brain metastases, tumor microenvironment characteristics, and prognosis.

**Materials and methods:**

A retrospective analysis was conducted on the clinical data of two ovarian cancer patients with brain metastases. One patient had a single brain metastasis and received comprehensive treatment including surgery, radiotherapy, chemotherapy, PD-1 inhibitor (tislelizumab), and PARP inhibitor (niraparib). The other patient had multiple brain metastases and was treated with chemotherapy combined with PD-1 inhibitor. Tissue microarray immunohistochemistry was used to detect the expression of immune markers (CD4, CD8, CD31, PD-L1, etc.) in primary tumors and brain metastases. Gene testing was performed to evaluate homologous recombination repair deficiency (HRD) status.

**Results:**

The patient with a single brain metastasis achieved complete response (CR) after comprehensive treatment, with significantly prolonged progression-free survival. The patient with multiple brain metastases showed significant short-term efficacy but died due to complications. Immunohistochemistry revealed higher expression of CD4, CD8, CD31, and PD-L1 in brain metastases compared to the primary tumor, suggesting increased T-cell infiltration and enhanced immune activity in the tumor microenvironment. Gene testing confirmed HRD positivity in the first patient, and maintenance therapy with PARP inhibitor combined with PD-1 inhibitor was effective.

**Conclusion:**

PD-1 inhibitors combined with chemotherapy and targeted therapy may provide a new treatment option for ovarian cancer patients with brain metastases, particularly those with single brain metastases who can achieve long-term survival through comprehensive treatment. The immune microenvironment characteristics of brain metastases (e.g., high PD-L1 expression, T-cell infiltration) may predict the efficacy of immunotherapy, but the prognosis for patients with multiple brain metastases remains poor. Further research is needed to explore the correlation between peripheral blood immune markers and treatment response in brain metastases.

## Introduction

From 1990 to 2021, the global burden of female-specific cancers, ovarian cancer is 0.30 million,Age-standardized rates of ovarian showed positive correlations with SDI,and additional risk factors are high BMI and occupational risks (1).

Brain metastases represent the most common intracranial malignant tumors, characterized by notably poor clinical prognosis and short patient survival. The incidence of brain metastases accounts for 20-40% of all metastatic cancer cases (2). To date, due to the protective effects of the blood-brain barrier, treatment options for brain metastases remain relatively limited, primarily relying on surgery and radiotherapy. However, the median overall survival following these therapies is still less than one year (3, 4). Additionally, patients with brain metastases are often excluded from clinical trials (5), and the insufficient research on brain metastases at both clinical and preclinical levels further delays the development of treatment options.

Immune checkpoints are critical components of the immune system and have provided new insights for cancer immunotherapy. The primary targets of immune checkpoint inhibitors are PD-1/PD-L1 (programmed cell death protein 1) on the surface of T cells. T cells are activated when their surface TCRs recognize MHC molecules on tumor cells. However, the PD-L1 protein on tumor cells can also bind to the PD-1 receptor on T cells, thereby inhibiting T cell activation. PD-1 inhibitors block the interaction between PD-L1 on tumor cells and PD-1 on T cells, disrupting the immunosuppressive tumor microenvironment. As a result, when the TCR on T cells binds to MHC molecules, the T cells are activated, awakening the immune system to attack tumor cells and increasing tumor cell death. The mechanism underlying the combined effect of chemoradiotherapy and immune checkpoint inhibitors mainly involves chemoradiotherapy-induced tumor cell apoptosis. Upon tumor cell death, a large number of antigens are released, activating immune cells (primarily CD8+ T cells). Meanwhile, immune checkpoint inhibitors block immunosuppression, thereby “releasing the brakes” on the immunogenicity of chemoradiotherapy. This enhances the phagocytic activity of immune cells against tumor cells.

## Materials and methods

### Case analysis and therapeutic efficacy evaluation

The first case is a 60-year-old female with primary high-grade serous ovarian carcinoma was first diagnosed in 2016. In February 2016, she underwent cytoreductive surgery for ovarian cancer at Shengjing Hospital of China Medical University. Postoperative paraffin pathology (Specimen 1) confirmed high-grade serous carcinoma in the omentum, bilateral adnexa, appendix, posterior bladder wall lesions, and intestinal surface lesions. Adenocarcinoma cells were identified in pleural effusion pathology. The patient was diagnosed with stage IVB ovarian cancer. Postoperatively, she received six cycles of paclitaxel plus carboplatin chemotherapy. Tumor recurrence with retroperitoneal lymph node metastases was detected in February 2017, prompting four cycles of liposomal doxorubicin combined with oxaliplatin chemotherapy. In October 2018, the patient developed headaches, and cranial MRI revealed a right parieto-occipital mass with perilesional edema (**Figure 1**), suggestive of metastatic or primary glioblastoma. She underwent craniotomy for metastatic lesion resection. Postoperative pathology (Specimen 2) confirmed ovarian cancer brain metastasis based on immunohistochemistry. Subsequent treatment included three cycles of albumin-bound paclitaxel plus cisplatin chemotherapy. In November 2019, tumor marker elevation prompted repeat enhanced cranial MRI (**Figure 2**), showing a new enhancing nodule near the right occipital sagittal sinus, suggesting recurrent metastasis with possible dural involvement. Gamma knife radiosurgery (**Figure 3**) was performed in December 2019 on two lesions: Right occipital sagittal sinus lesion: 28.7 mm × 23.4 mm × 35.9 mm, conformal extended field, target volume 18.49 cm³, 26 isocenters, 50% isodose line, peripheral dose 11.0 Gy, central dose 22.0 Gy. Right occipital surgical bed recurrence: 28.8 mm × 17.0 mm × 25.0 mm, conformal extended field, target volume 12.41 cm³, 13 isocenters, 50% isodose line, peripheral dose 11.0 Gy, central dose 22.0 Gy. The patient recovered well postoperatively. However, recurrence was observed in July 2020, with enlargement of the right parieto-occipital lesion and dural involvement (**Figure 4**). A second craniotomy was performed, and pathology (Specimen 3) confirmed recurrent ovarian cancer metastasis. Genetic testing (BGI Genomics, Shenzhen) in August 2020 revealed homologous recombination repair deficiency, indicating sensitivity to PARP inhibitors. From August to November 2020, she received four cycles of albumin-bound paclitaxel combined with bevacizumab (100 mg/4 mL, Qilu Pharmaceutical) and tislelizumab (200 mg, BeiGene). Follow-up MRI in November 2020 showed postoperative changes and a right occipital softening lesion (**Figure 5**), achieving complete response (CR) per RECIST 1.1 criteria. Maintenance therapy with oral niraparib (200 mg daily) and intravenous tislelizumab (200 mg every 3 weeks) was initiated in December 2020, with no recurrence observed during follow-up.

**Figure 1:**
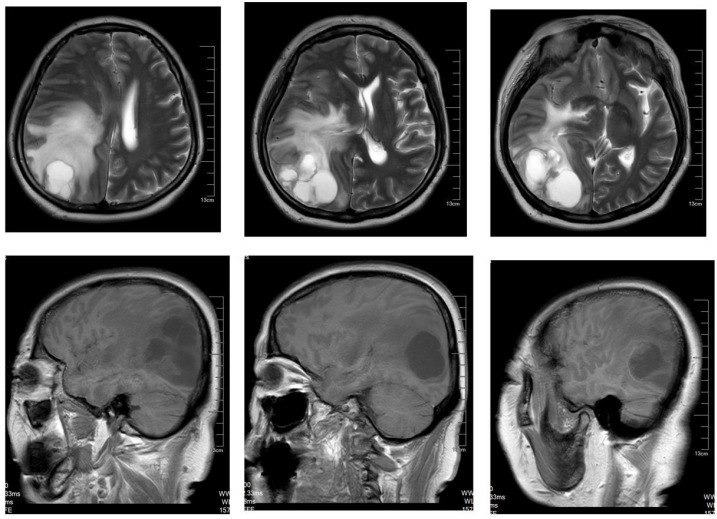
Right parietal-occipital lobe space-occupying lesion with surrounding brain parenchymal edema.

**Figure 2:**
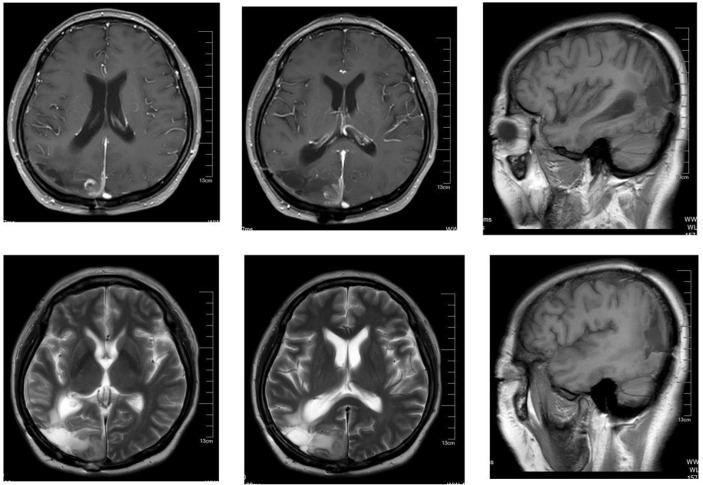
Contrast-enhanced MRI of the head.

**Figure 3:**
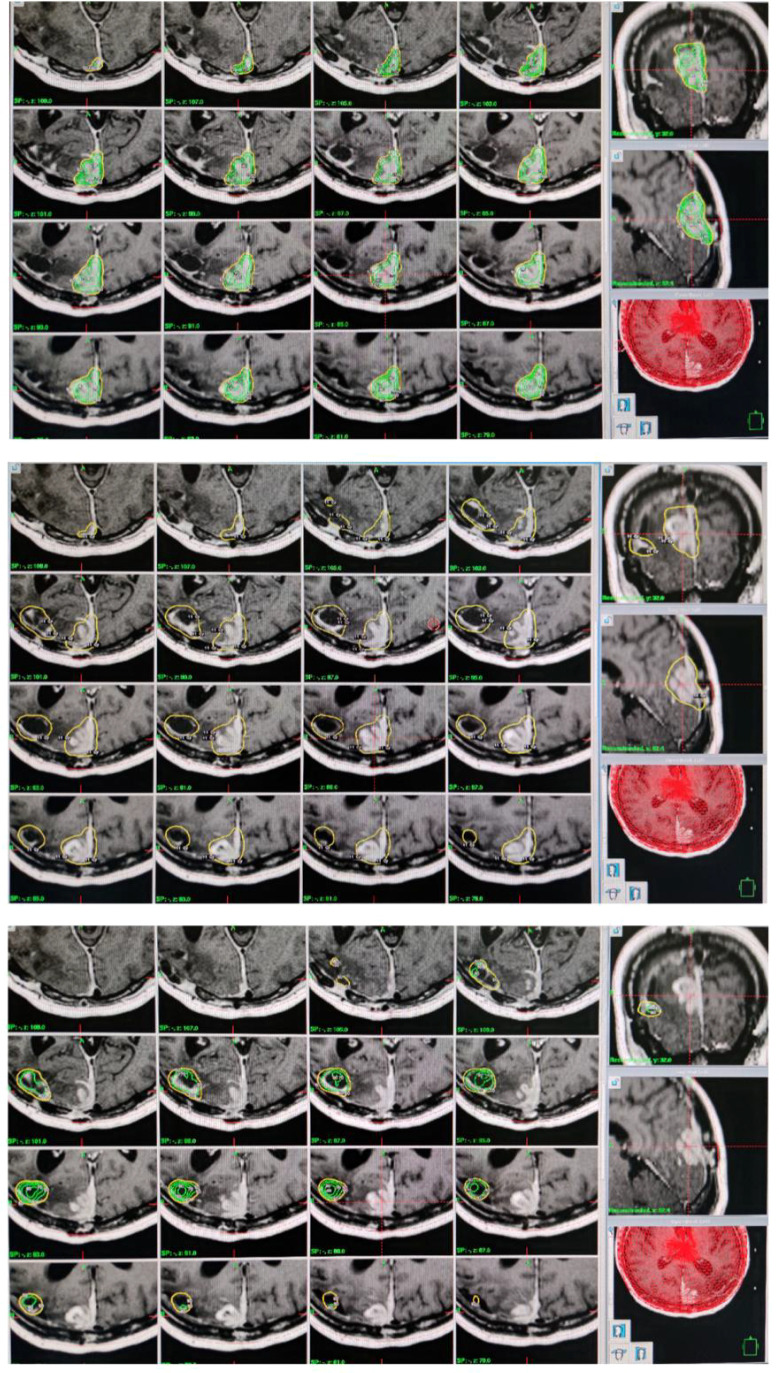
Gamma knife radiosurgery for two intracranial lesions.

**Figure 4:**
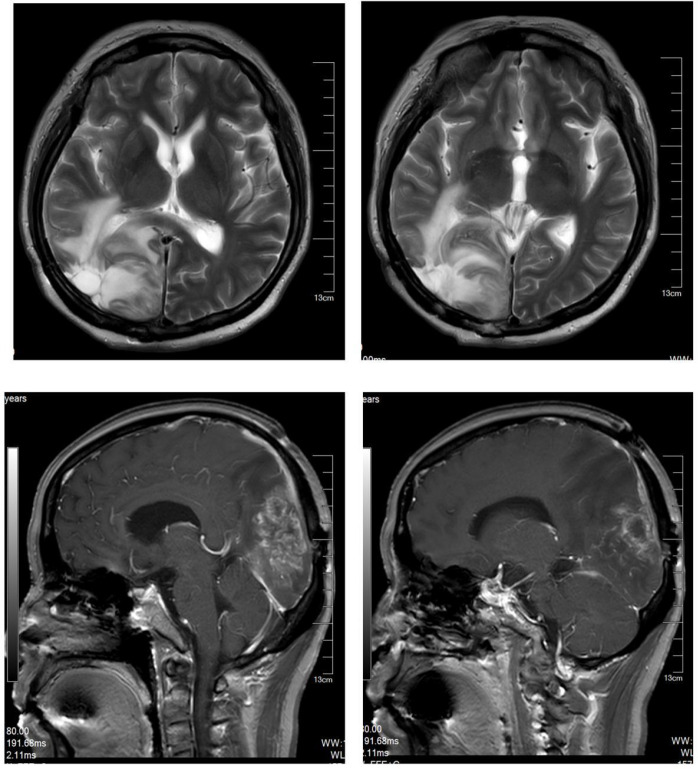
In the right parieto-occipital region adjacent to the sagittal sinus, the enhancing nodule has increased in size compared to previous imaging, with expansion of the adjacent meningeal involvement.

**Figure 5:**
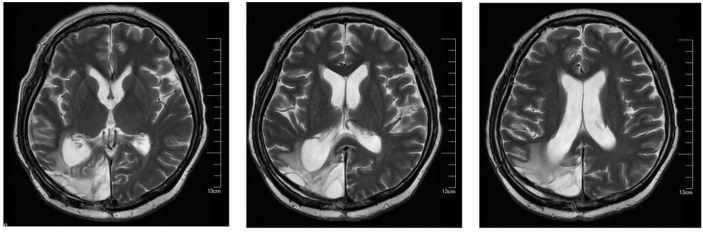
Postoperative intracranial changes with encephalomalacia in the right parieto-occipital lobe.

The second case is a 56-year-old female with ovarian cancer brain metastasis received chemotherapy combined with PD-1 inhibitors without surgery or radiosurgery due to multiple metastatic lesions. The patient was initially diagnosed in May 2018 with stage IVB ovarian cancer based on pleural effusion cytology (adenocarcinoma) and PET-CT findings (right adnexal mass, peritoneal metastases, and pleural effusions). Neoadjuvant chemotherapy with paclitaxel (135–175 mg/m², Yangtze River Pharmaceutical Group) plus carboplatin (AUC = 5) was administered for two cycles, followed by interval cytoreductive surgery in May 2018. Postoperative pathology confirmed high-grade serous carcinoma in bilateral adnexa, appendix, omentum, and rectal surface. Six cycles of adjuvant paclitaxel-carboplatin chemotherapy were completed. In May 2020, recurrence with multiple brain metastases was detected via PET-CT and cranial MRI (**Figure 6**). The patient received three cycles of albumin-bound paclitaxel (400 mg, CSPC Pharmaceutical Group), cisplatin (40mg, Qilu Pharmaceutical), and tislelizumab (200mg, BeiGene). Post-treatment MRI showed reduced tumor burden and edema (**Figure 7**), with symptomatic improvement. Two additional chemotherapy cycles were administered. However, the patient died of hypertensive intracranial hemorrhage two weeks after the last cycle. No autopsy was performed, and the exact etiology of hypertension or potential leptomeningeal involvement remains unclear. Despite this, significant tumor regression was observed after three cycles of PD-1 inhibitor-based therapy.

**Figure 6:**
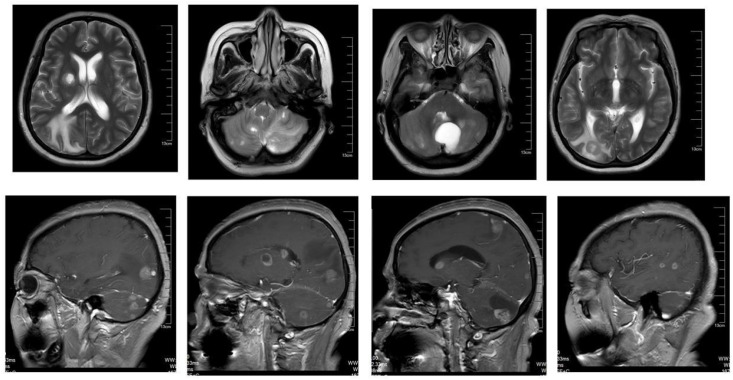
Head PET-CT and contrast-enhanced MRI reveal multiple intracranial metastases.

**Figure 7:**
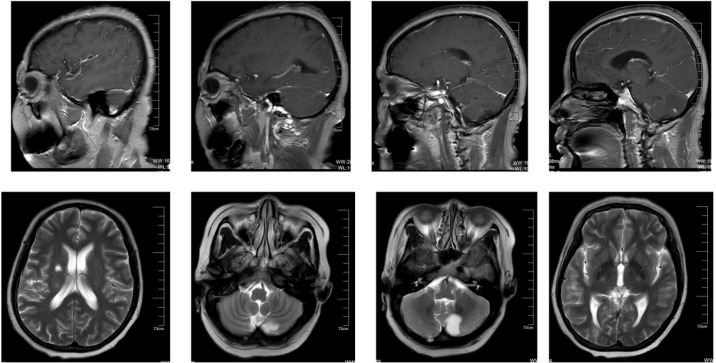
After 3 cycles of intravenous chemotherapy, a follow-up contrast-enhanced MRI of the head showed a decrease in both size and number of multiple intracranial metastases compared to previous imaging.

## Results

### Multiplex immunohistochemistry results

For the first patient with brain metastasis, multiplex immunohistochemistry was used to detect the expression of cell surface antigens (CD4, CD8, CD31, CD25, CTLA-4, and PD-L1) in two brain metastasis specimens and the primary ovarian cancer specimen (**Figure 8**). Data were analyzed using Prism 9 (**Figure 9**). The results showed that CD4, CD8, CD31, and PD-L1 expression levels were significantly elevated in the second brain metastasis specimen compared to the primary tumor, suggesting that not all brain metastases may be inherently resistant to PD-1 inhibitor therapy. **Figure 9** highlights that PD-L1 expression was notably higher than other markers.

**Figure 8:**
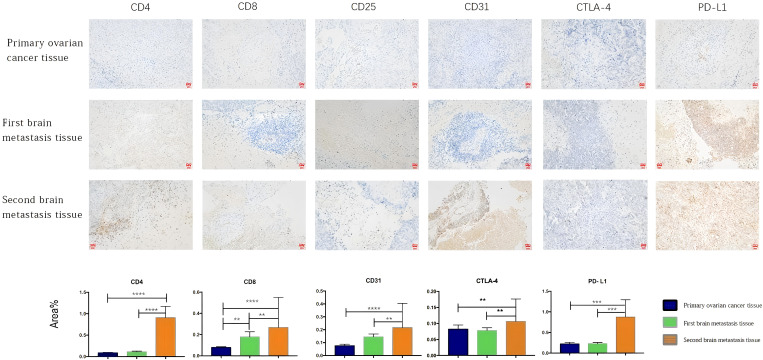
Expression profile of surface antigens CD4, CD8, CD31, CD25, CTLA-4, and PD-L1 on tumor-infiltrating immune cells (TILs). ***: p < 0.001; ****: p < 0.0001; *****: p < 0.00001.

**Figure 9:**
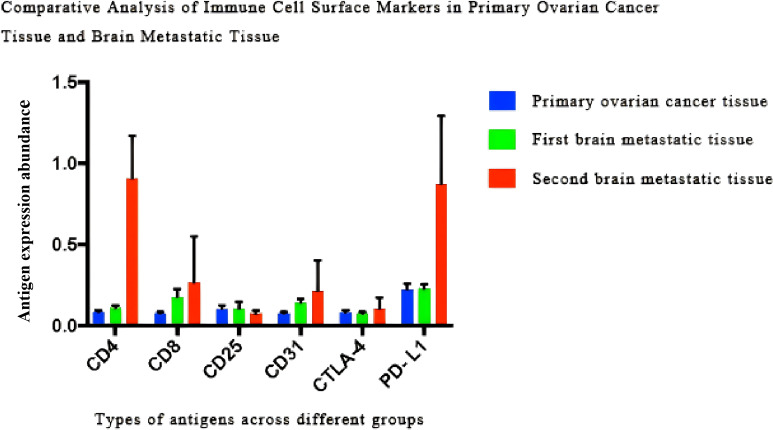
Case 1: comparative analysis of surface markers on tumor-infiltrating immune cells (TILs) in primary ovarian cancer tissue and brain metastatic tissue.

CD31, a vascular endothelial cell marker, reflects tumor angiogenesis. Its detection in the patient’s brain metastases (with higher expression in both metastases than the primary tumor) may explain the efficacy of combining chemotherapy, bevacizumab, and PD-1 inhibitors.

CD4 and CD8, markers of helper T cells and cytotoxic T cells, exhibited varying degrees of expression across specimens. Their most pronounced expression in the second brain metastasis suggests the highest infiltrating T-cell count in this sample, surpassing even the primary tumor.

Genetic testing post-second brain metastasis surgery revealed negative BRCA1/2 germline variants (**Figure 10**, **11**) but an HRD-positive score (**Table 1**), confirming homologous recombination deficiency (**Table 2**). Maintenance therapy with PARP inhibitors combined with PD-1 inhibitors achieved durable efficacy.

**Figure 10:**
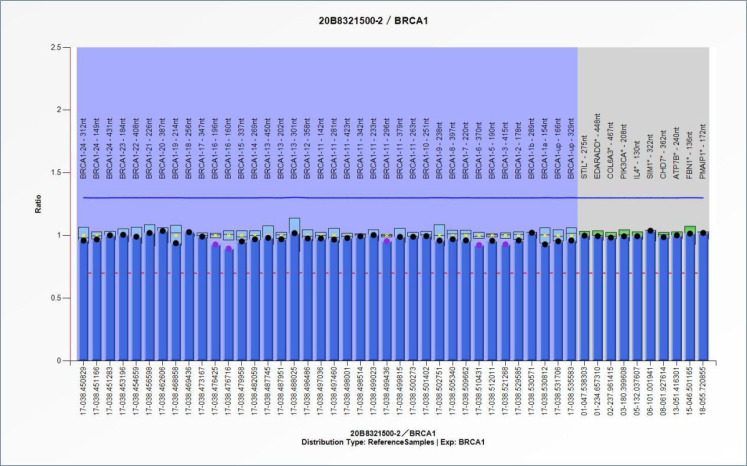
In Case 1, genetic testing perfor`med after the initial surgery for brain metastasis revealed no detectable BRCA1/2 germline variants.

**Figure 11:**
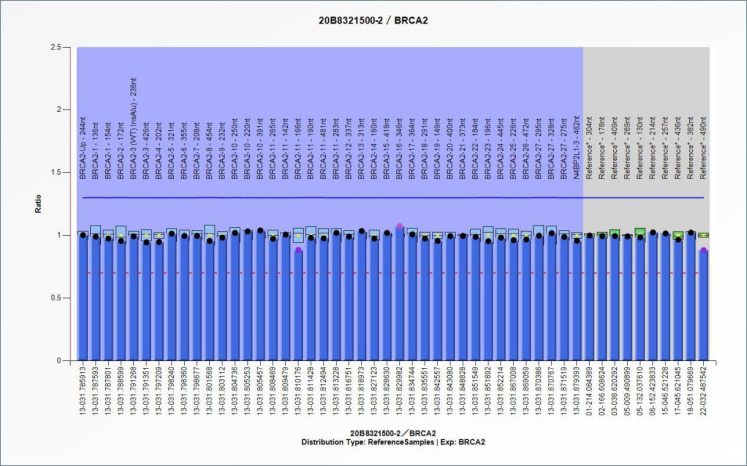
In Case 1, genetic testing performed after the second surgery for brain metastasis revealed no detectable BRCA1/2 germline variants.

**Table 1:** HRD score calculation.

LOH	TAI	LST	Purity/Ploidy adjustment	HRD score
19	16	29	1.75	36.94

**Table 2:** Comprehensive HRD status.

HRD score	BRCA1/2 mutation status	HRD comprehensive status
Positive	Negative	Homologous Recombination Deficiency (HRD) Confirmed

Case Two involved a patient with multiple ovarian cancer brain metastases. While chemotherapy combined with PD-1 inhibitors demonstrated rapid short-term efficacy, the high risk of hypertensive cerebral hemorrhage posed challenges for long-term survival management.

## Discussion

Brain metastases in most ovarian cancer patients are not incidental events but rather occur predominantly in later-stage disease. In this study, all mentioned patients presented with stage IV ovarian cancer as their initial diagnosis, with hematogenous metastasis being the primary mode of spread. This suggests that patients with distant metastases at initial diagnosis represent a high-risk population for developing brain metastases.

Ovarian cancer is a rare cause of brain metastasis. Development of brain metastasis among older patients and lower KPS score correlate with less favorable prognosis, and the number of intracranial metastatic lesions is an equally critical prognostic factor—solitary brain metastases exhibit far better responses to PD-1 inhibitor-based therapy compared with multiple lesions, a difference closely tied to the distinct tumor microenvironment (TME) characteristics between the two conditions. The more prolonged survival after using multimodality treatment for brain metastasis is important due to potential impact on management of brain metastasis in future (6). The blood–brain barrier (BBB) is also a key factor in BM; dysregulation of cell junctions, tumor microenvironment and loss of function of microglia leads to BBB disruption ultimately resulting in BM (7), and the degree of BBB impairment differs notably in solitary versus multiple brain metastases—solitary lesions typically present with localized, moderate BBB disruption that facilitates the targeted infiltration of PD-1 inhibitors and activated T cells into the tumor site without widespread intracranial immune disturbance, while multiple metastases cause extensive, disorganized BBB breakdown that not only limits the effective delivery of immunotherapeutic agents to individual lesions but also induces a systemic immunosuppressive intracranial microenvironment. Such TME differences exert a profound influence on therapeutic efficacy: solitary brain metastases feature elevated levels of CD4+, CD8+ T cells, CD31 and PD-L1 in the TME, with abundant tumor-infiltrating lymphocytes and enhanced local immune activity that synergizes with PD-1 inhibitors to reverse T-cell suppression and amplify anti-tumor immune responses, whereas multiple brain metastases harbor a more heterogeneous and immunosuppressive TME with inconsistent immune cell infiltration and PD-L1 expression across lesions, blunting the anti-tumor effects of PD-1 inhibitors and leading to suboptimal therapeutic outcomes. In addition, Non-proteincoding transcripts bearing 200 base pairs known as long non-coding RNAs (lncRNAs) play a role in a variety of molecular mechanisms, including cell differentiation, apoptosis and metastasis (8).BRCA1 and BRCA2 gene mutations, as well as the expression of androgen receptors in the primary tumor, are emerging risk and prognostic factors which could allow one to identify categories of patients at greater risk of BMs, who could benefit from a tailored follow-up. Hopefully, novel therapeutic avenues are being explored, like PARP inhibitors and immunotherapy, based on our improved knowledge regarding tumor biology and the TME-specific features of solitary versus multiple brain metastases, but further investigation is warranted (9).

The PD-1 immune checkpoint primarily functions within the tumor microenvironment by binding its ligand to suppress T-cell activity (10). First, the permeability of intracranial tumor vasculature is lower than that of extracranial tumor vasculature (11), limiting the delivery of sufficient PD-1 checkpoint inhibitors to the brain to relieve T-cell inhibition. Second, effective intracranial anti-tumor immunity relies on the release of tumor antigen-dependent T cells. However, since brain metastases originate from intracranial tumors, they may fail to adequately drain tumor antigens to regional lymph nodes, thereby impairing the initiation of robust T-cell responses against tumor-specific antigens.

Cervical lymph nodes serve as the lymphatic drainage site for intracranial lymphatic systems and are induced to proliferate T cells following intracranial tumor growth (12). Characteristics of tumor-draining lymph nodes may significantly impact immune responses. Studies show that as tumor antigens accumulate in cervical lymph nodes, suppressive lymphocyte numbers increase markedly while CD8+ T-cell counts decrease, suggesting cervical lymph nodes are more prone to inducing tumor tolerance compared to other lymph nodes (12). Additionally, recent discoveries of lymphatic vessels in the dura mater contrast with the absence of such structures in brain parenchyma (13)—objective limitations constraining intracranial immune responses.

Numerous studies confirm the presence of tumor-infiltrating lymphocytes (TILs) in brain metastases (14–17). However, some reports indicate lower TIL levels in brain metastases compared to primary tumors or extracranial metastases (18–21), conflicting with our immunohistochemical findings. Our data reveal that lymphocytic infiltration in brain metastases exceeds that in primary tumors, particularly in the second metastasis specimen. Notably, several studies associate TIL infiltration in brain metastases with patient prognosis (14, 15, 17, 20, 24), aligning with our observations.

However, when brain metastases develop, leakage in the blood-brain barrier may allow increased doses of PD-1 inhibitors to directly reach tumor sites. Emerging evidence suggests therapeutic antibodies could be transported into the brain by activated T cells—for instance, PD-1 checkpoint inhibitors activate T cells in peripheral blood, which then carry the antibodies into brain metastases (25). Increased barrier permeability correlates with greater leakage of therapeutic antibodies and T cells into the brain.

Immune checkpoints critically regulate effector T-cell function and anti-tumor immunity. Differences in checkpoint expression between intracranial and extracranial tumors may explain divergent treatment responses. While PD-1/PD-L1 and CTLA-4 are clinically relevant checkpoints, there remains a lack of evidence comparing their expression patterns in brain metastases versus primary tumors.

Emerging studies suggest that T cells play a critical role in propagating immune therapy efficacy to intracranial metastases (24). Effective T-cell trafficking to tumors is pivotal for ensuring therapeutic outcomes. Research indicates that intracranial tumors induce T-cell proliferation predominantly in cervical and lumbar lymph nodes (LN), with T-cell infiltration into brain metastases originating from cervical LN being 2.5-fold higher than from inguinal LN (26). While cervical LN are implicated in promoting tumor tolerance (12), peripheral PD-1 inhibition may enhance CD8+ T-cell infiltration into intracranial tumors by releasing antigen-specific T-cell suppression.

Our multiplex immunohistochemistry results revealed markedly elevated CD4, CD8, CD31, and PD-L1 expression in both brain metastases, particularly in the second recurrence, surpassing levels in the primary tumor—a finding inconsistent with prior literature (18–23). This challenges the assumption that all brain metastases are inherently resistant to PD-1 inhibitors. Tumor heterogeneity and recurrence driving differential immune profiles. These observations raise questions about integrating chemotherapy with immunotherapy for ovarian cancer brain metastases. However, logistical barriers—such as limited tumor biopsy access—prompt exploration of peripheral blood biomarkers (e.g., T-cell activation status) as proxies for intratumoral TIL activity and PD-1 inhibitor responsiveness. Such hypotheses require validation through translational and clinical trials.

The two cases described here are typical examples of ovarian cancer brain metastases. The first patient presented with a solitary brain metastasis, allowing time to employ a multimodal treatment strategy, including surgery, radiation, chemotherapy, immunotherapy, and targeted therapy. The presence of brain metastases may indicate compromised blood-brain barrier integrity, offering opportunities to explore diverse therapeutic approaches tailored for ovarian cancer. The second case involved multifocal brain metastases; while PD-1 inhibitor-based immunotherapy demonstrated rapid short-term efficacy, the inherent risk of hypertensive cerebral hemorrhage poses significant challenges for long-term survival.

Between January 1, 2016, and December 31, 2021, we treated 15 ovarian cancer patients with brain metastases. Regrettably, most have succumbed to the disease, leaving only two survivors—both benefiting from comprehensive therapy for solitary brain metastases.

Based on these clinical outcomes and preclinical data: Number of intracranial lesions strongly correlates with prognosis in ovarian cancer brain metastases. Comprehensive therapy is indispensable for managing this condition. PD-1 inhibitors play a meaningful role in both upfront and maintenance therapies, emerging as a promising addition to the multidisciplinary treatment paradigm for ovarian cancer brain metastases.

## Data Availability

The raw data supporting the conclusions of this article will be made available by the authors, without undue reservation.
